# Supplementary material for: Virtual Environment Rehabilitation for Patients with Motor Neglect Trial (VERMONT): A Single-Center Randomized Controlled Feasibility Trial
Source: Brain Sci. 2021 Apr 6;11(4):464. doi: 10.3390/brainsci11040464 (PMC8067499; doi:10.3390/brainsci11040464)
Supplement: Supplementary file 1 [file brainsci-11-00464-s001.pdf]

Table S1. Distance walk per session per subject at Baseline and at Week 2

CONTROL	Subject No		0	1	2	3	4	5	mean
	6	**Baseline**	67.5	115.9	150.8	234.0			142.1
		**2 week**	66.0	136.0	156.3	156.3			128.6
	9	**Baseline**	20.2	117.9	45.4	273.8			114.3
		**2 week**	37.8	3.4	155.9	195.8	184.9		115.6
	11	**Baseline**	64.9	82.9	72.8	94.3	80.3		79.0
		**2 week**	108.9	42.9	74.8	12.9	42.9		56.5
	18	**Baseline**	190.2	164.9	239.5	298.0	357.4	365.8	269.3
		**2 week**	176.8	181.9	168.3	174.4			175.4
		**Baseline**	85.7					238.5	151.2
		**2 week**	97.4					139.6	139.6

INTERVENTION			0	1	2	3	4	5	mean
	1	**Baseline**	67.3	76.2	74.2	64.0	74.6		71.2
		**2 week**	96.0	117.6	119.9	119.0	108.9		112.3
	2	**Baseline**	32.5	64.2	72.4	84.6	98.1		70.4
		**2 week**	74.5	86.3	89.0	90.6	90.5		86.2
	3	**Baseline**	34.9	47.0	51.4	68.4	74.2		55.2
		**2 week**	18.2	116.0	133.9	128.8	171.7		113.7
	13	**Baseline**	71.0	87.0	105.3	100.5	136.4		100.0
		**2 week**	98.1	123.7	175.0	189.2	191.2		155.4
	14	**Baseline**	138.2	163.8	186.9	205.5	212.1		181.3
		**2 week**	160.8	203.8	202.6	210.2			194.3
	19	**Baseline**	139.1	162.0	164.3	156.1	170.1		158.3
		**2 week**	139.5	196.4	194.4	184.6	205.7		184.1
		**Baseline**	86.8				127.6		106.1
		**2 week**	97.8				163.5		141.0
